# Hierarchical Porous Polyamide 6 by Solution Foaming: Synthesis, Characterization and Properties

**DOI:** 10.3390/polym10121310

**Published:** 2018-11-27

**Authors:** Liang Wang, Yu-Ke Wu, Fang-Fang Ai, Jie Fan, Zhao-Peng Xia, Yong Liu

**Affiliations:** 1 School of Textiles, Tianjin Polytechnic University, No.399 Binshui West Road, Xiqing District, Tianjin 300387, China; 13388096568@163.com (Y.-K.W.); 13032255632@163.com (F.-F.A.); fanjie@tjpu.edu.cn (J.F.); xiazhaopeng@tjpu.edu.cn (Z.-P.X.); 2 Key Laboratory of Advanced Textiles Composites of Ministry of Education, Tianjin Polytechnic University, Binshui West Road 399, Tianjin 300387, China

**Keywords:** foams, polyamide, crystalline, thermal conductivity, mechanical property

## Abstract

Porous polym er materials have received great interest in both academic and industrial fields due to their wide range of applications. In this work, a porous polyamide 6 (PA6) material was prepared by a facile solution foaming strategy. In this approach, a sodium carbonate (SC) aqueous solution acted as the foaming agent that reacted with formic acid (FA), generating CO_2_ and causing phase separation of polyamide (PA). The influence of the PA/FA solution concentration and Na_2_CO_3_ concentration on the microstructures and physical properties of prepared PA foams were investigated, respectively. PA foams showed a hierarchical porous structure along the foaming direction. The mean pore dimension ranged from hundreds of nanometers to several microns. Low amounts of sodium salt generated from a neutralization reaction played an important role of heterogeneous nucleation, which increased the crystalline degree of PA foams. The porous PA materials exhibited low thermal conductivity, high crystallinity and good mechanical properties. The novel strategy in this work could produce PA foams on a large scale for potential engineering applications.

## 1. Introduction

Polyamide 6 (PA6), also known as Nylon 6, is widely known for its high impact resistance, good toughness, abrasion resistance and strength. Due to its excellent physical properties, PA6 is widely used in industry, for example, as textile fibers and engineering polymer composites [1]. It is expected that PA6 can be processed into lightweight products that are used in the field of insulation and cushioning.

Porous polymer materials have attracted wide attention from both industry and academia. Depending on the application, the porous material must meet specific requirements. Thus, great effort has been invested in the manipulation of their properties. Besides the material composition, the porous structure plays a crucial role when it comes to the tailoring of porous materials [2]. Traditional polymer foams, for example, expanded polystyrene, are produced from polymer melts and blowing agents [3]. They are usually used in fields such as packaging, insulation, and impact protection. Supercritical fluid foaming technology is developed to manufacture microcellular foams with the cell size in the order of 1–100 μm. In this process, gas diffuses into polymers and then bubbles nucleate in a gas–polymer system at a high temperature [4]. Thermoplastic microcellular foams with improved properties are obtained for possible engineering applications [5].

Recently, alternative strategies have been designed to process polymers into functional porous materials. High-internal-phase emulsions polymerization used water-in-oil emulsions as templates to construct cellular structures in subsequent synthesized polymers [6,7,8]. Anionic polymerization realized a porous structure by controlling the phase separation and growth of spherulitic domains during polymerization [9]. Foam-like cryogels were produced by sublimating ice templates from frozen polymer gels via freeze-drying [10,11]. Nanoporous aerogels were also fabricated from wet gels by sol-gel chemistry via supercritical drying technique [12,13]. The phase inversion method generated pores in wet phase using solvents exchange and then porous monoliths were obtained after drying at ambient pressure [14,15]. However, supercritical drying and freeze-drying are slow and energy-consuming, making the large-scale production of aerogels very expensive and risky. Solvent exchange in phase inversion is not environmentally friendly and is also time-consuming. The polymerization method usually suffers from complicated processing. New strategies with high efficiency and low cost are desirable to produce novel porous materials on a large scale. 

In the present work, we report an efficient, low-cost and template-free method for manufacturing polyamide (PA) foams. In this process, Na_2_CO_3_ aqueous solution, used as a foaming agent, was injected into a PA/formic acid (FA) solution. The reaction between Na_2_CO_3_ and FA induced phase separation of PA and formed the cell walls of porous materials. Meanwhile, CO_2_ was generated, and bubbles were nucleated in polymer solution by creating a thermodynamic instability. The influence of foaming parameters, that is, concentration of PA/FA solution and Na_2_CO_3_ aqueous solution, on microstructures, crystallinity, compressive mechanical properties and thermal conductivity were investigated.

## 2. Materials and Methods 

### 2.1. Material

Polyamide 6 (PA6) with a molecular weight of 20,000 was brought from Ube Industries (Osaka, Japan). Sodium carbonate (SC) and formic acid (FA) were produced by Tianjin Fengchuan chemical reagents (Tianjin, China). All chemical reagents were used as received.

### 2.2. Preparation of PA Foams

**Figure 1:**
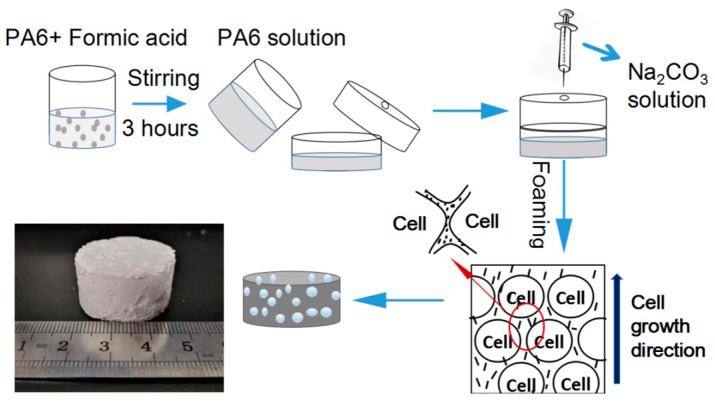
Scheme of preparation procedure of porous PA materials.

The preparation process of PA foams is illustrated in Figure 1. Nylon-6 pellets were dissolved in anhydrous formic acid with a desirable concentration through magnetic stirring at room temperature for 3 h. Meanwhile, sodium carbonate (SC) solutions with desirable content were prepared by dissolving SC particles in deionized (DI) water. Then, 6 mL of transparent PA/FA solution were transferred into a cylinder mold with a diameter of 30 mm and height of 20 mm. Subsequently, excessive SC solutions were injected into the mold through a syringe. Large amounts of CO_2_ were generated by the neutralization reaction and bubbles nucleated in the viscous solution. Along with the PA molecules separated from the solvent, the bubbles grew, foaming the porous structure. The obtained porous materials were washed with DI water four times and dried in an oven at a temperature of 60 °C for 6 h. Samples prepared were named by PA concentration followed by blow agent solution (SC) percentage according to the processing parameters, for example, 12PA-3SC.

### 2.3. Characterizations

A field emission scanning electron microscope (ZEISS GeminiSEM 500, Oberkochen, Germany) was used to characterize the macroporous structures of the sample. Prior to observation, all the samples were cryo-fractured by immersing them in liquid N_2_ (−196 °C), and were then sputtered with gold to ensure sufficient conductivity. Pore diameters of foams were measured using Image J software.

Differential scanning calorimetry (DSC) was performed using a 200F3 equipment (Netzsch, Ahlden, Germany) following the procedure described below. A 10 mg tested sample was loaded in an aluminum pan and first heated from 25 °C to 280 °C at a heating rising ramp of 10 °C/min. The pan was held at 280 °C for 5 minutes and then cooled to 25 °C at the same temperature ramp. The crystallinity (*X*_c_) of porous PA6 was calculated according to the first melting curve using the following equation:

$$X_{c}\left(\%\right)=\frac{\Delta H_{m}}{\Delta H_{c}}\times 100$$

where ∆*H*_c_ for 100% crystalline PA6 is 188 J/g [16].

Wide-angle X-ray diffraction (WAXD) patterns were recorded in a D8 Discover X-ray diffractometer (Bruker, Karlsruhe, Germany) with CuKα radiation (*λ* = 0.154 nm).

The bulk densities (*ρ*_b_) of PA foams were calculated by the division of mass to volume of cylindrical samples. Five samples were used to evaluate each composition.

The thermal conductivity of sample was measured using a TPS 2500S equipment (Hot Disk, Uppsala, Sweden) based on ISO 22007-2.2. Prior to testing, two prepared cylinder samples with flat bottoms were prepared by slight polishing. A thermo sensor probe was placed between the two samples during the tests.

Compression tests were performed using a universal testing machine (Hongda, Beijing, China) with a load cell of 5 kN. The crosshead rate and maximum strain were set to 1 mm/min and 60%, respectively. To determine the elastic modulus, compression tests with intermittent unloading (to zero force) and reloading were conducted additionally at ambient condition (20 °C and 65% humidity). Five replicas were tested for each composition. 

## 3. Results and Discussion

### 3.1. Morphologies

**Figure 2:**
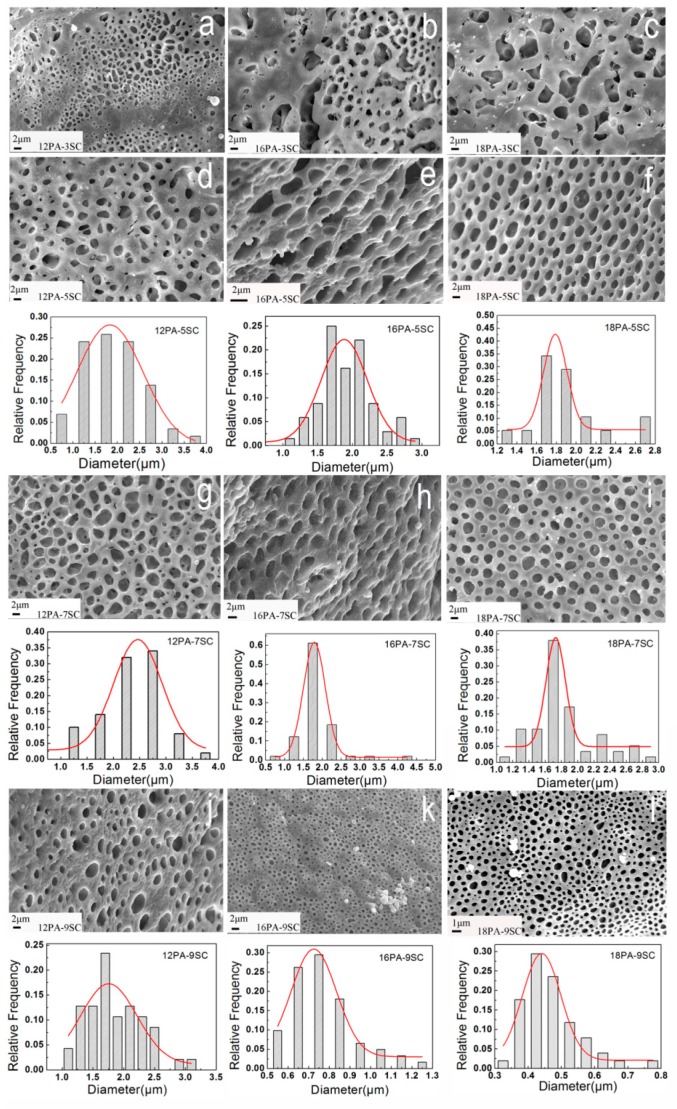
SEM images of PA foams with different compositions and their corresponding pore’s size distribution: (**a**) 12PA-3SC; (**b**) 16PA-3SC; (**c**) 18PA-3SC; (**d**) 12PA-5SC; (**e**) 16PA-5SC; (**f**) 18PA-5SC; (**g**) 12PA-7SC; (**h**) 16PA-7SC; (**i**) 18PA-7SC; (**j**) 12PA-9SC; (**k**) 16PA-9SC; (**l**) 18PA-9SC.

Each sample was characterized at a similar position and the corresponding SEM images of PA foams are shown in Figure 2. It was found that the microstructures of three-dimensional PA foams depended on two factors, including the amount of CO_2_ produced by reaction of Na_2_CO_3_ (SC) and formic acid (FA) and the separation rate of PA molecules from FA. Irregular porous structures were generated when 3 wt % of SC solution was used as a blowing agent, as seen in Figure 2a–c. This was because the low foaming power resulted from the low concentration of SC. With the increase of SC concentration, cellular structures appeared and the pores decreased in dimensions. For instance, when the concentration of SC increased from 5% to 9% with fixed 16 wt % PA, the average cell diameter of corresponding samples reduced from 2.2 to 0.75 μm (Figure 2e,k). This change of microstructure could be attributed to two factors: On one hand, higher concentration of SC solution generated larger amounts of CO_2_, thereby leading to a high pressure in the bubble, which could refine the cellular structure and reduce the pore size [17]. On the other hand, nucleation sites for polymers during the foaming stage increased due to the heterogeneous nucleation effect of sodium salts generated from the neutralized reaction. This limited the expansion of bubbles and therefore made the obtained PA foams have smaller pores with even distribution [18]. However, when the concentration of SC was 9%, excessive pressure in the mold intensified the combination of air bubbles, causing defects in the cellular structure and uneven distribution of pore dimension, as seen in Figure 2j.

Sample 12PA-5SC showed an irregular porous structure (Figure 2d). When a proper fraction of SC solution was used, a higher PA concentration led to greater viscosity of the solution. This was able to enhance homogeneous nucleation, generating a more regular internal structure (Figure 2e,f). Moreover, the increase of viscosity may affect the expansion of bubbles and retard the growth of foams, resulting in smaller pores and thicker cell walls [19].

In general, both solution viscosity and foaming rate could affect the morphologies of the prepared PA foams. The released CO_2_ amount and the viscosity of solution should be adjusted to obtain the optimal cellular structure. Both PA concentration and SC content had a critical influence. 

**Figure 3:**
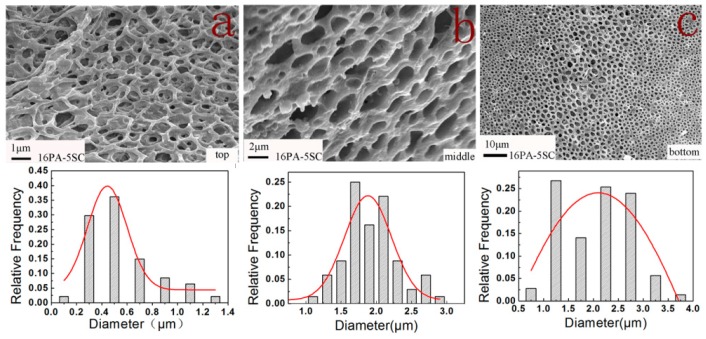
SEM images of the structural changes and pore size distribution along the foaming direction: (**a**) top, (**b**) middle and (**c**) bottom part of 16PA-5SC.

The structural changes of PA foams along the foam growth direction were studied by taking sample 16PA-5SC as a representative. Figure 3 shows the morphologies of three positions of bulk sample from bottom to top (foaming direction). The average pore diameter, pore size distribution and cell wall thickness of the porous material had a hierarchical change along the direction of foaming. The close pore percentage was relatively high at the beginning of foaming, resulting in an average pore diameter of ~2 μm in the bottom of the sample (Figure 3c). As the foam grew, the mold’s space was progressively occupied. Continuous CO_2_ release increased the pressure on the mold, which increased the open cell content and decreased the mean pore diameter to 0.5 μm (Figure 3a) [20].

### 3.2. DSC Analysis

**Figure 4:**
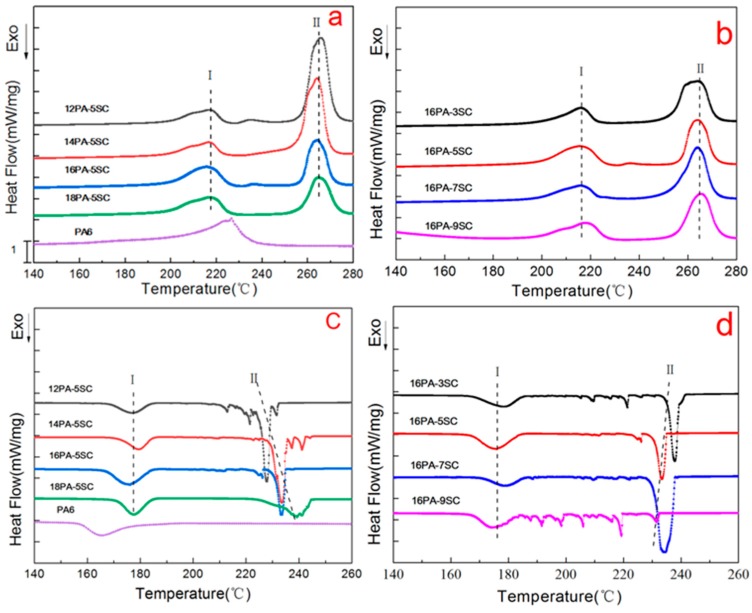
Differential scanning calorimetry (DSC) patterns of raw PA6 and PA foams. (**a**,**b**): first melting scans; (**c**,**d**): first cooling scans.

The effect of processing parameters on crystalline properties was studied by DSC. The first heating curve and the first cooling pattern are shown in Figure 4. Information such as crystallinity (*X*), crystallization temperature (*T*_c_) and melting temperature (*T*_m_) provided by DSC analysis are included in Table 1. PA6 polymer showed a single melting peak at 226.3 °C. However, the prepared PA foams in this work exhibited a multiple melting phenomenon. The sodium salts played a role of heterogeneous nucleation and crystal nuclei formed rapidly at a high temperature (230–240 °C). Crystals grew by a polymer chain segments arrangement on the surface of nuclei, making the PA foams have a melting peak at ~265 °C. Another melting peak was located at the low temperature side (~216 °C), which was lower than the *T*_m_ of raw PA6, indicating that the foaming process was not beneficial for lamellae stacking. This was possible due to the generated CO_2_, which prevented the arrangement and stacking of polymer chain segments. In addition, the prepared PA foams had much higher crystalline degree than raw PA6, as shown in Table 1, resulting from the heterogeneous nucleation effect of generated sodium salts. 

By increasing SC solution concentration to 7%, the crystallinity increased. Sodium salts generated from neutralization in the PA matrix played an important role for heterogeneous nucleation. The nucleation sites increased and the crystallizing rate also increased, which shortened the time for segmental rearrangement to a certain extent [21]. However, when the SC concentration increased to 9%, the crystallinity dropped. Large quantities of sodium salt caused competition for nucleating sites, preventing heterogeneous nucleation, as seen in the crystallizing pattern in Figure 4d [22,23]. In addition, 16PA-9SC exhibited multiple crystallizing peaks. Rapid and massive nucleation inhibited the growth of crystals and produced a large amount of non-perfect crystals in the polymer matrix. 

When the PA concentration increased from 12% to 16%, no significant change was observed of the crystallinity in the foams. Higher crystallinity was obtained by further increasing the PA concentration to 18%. Nucleation and diffusion were the two factors determining crystallization behaviors of PA foams [24]. With an increase of PA concentration, the nucleation rate was increased. Meanwhile, the diffusion speed and growth rate of the crystal nucleus slowed down due to increased viscosity of solutions [25]. Therefore, PA molecular chain segments had sufficient time to rearrange, resulting in a significant increase of the crystallinity.

### 3.3. WAXD Analysis

**Figure 5:**
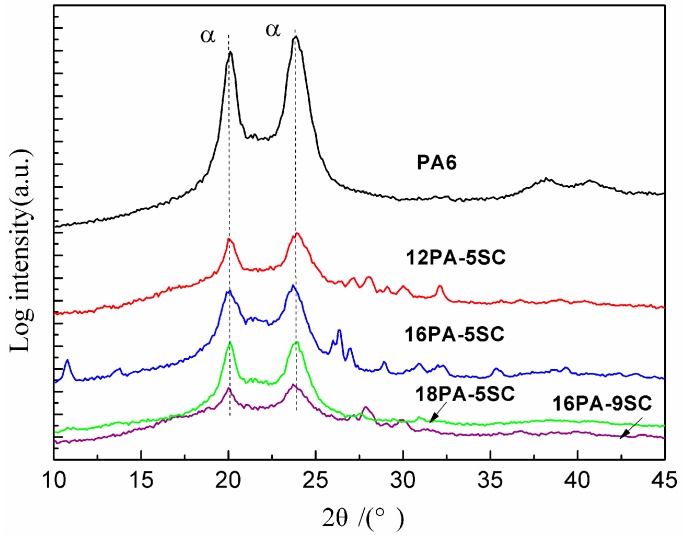
XRD patterns of raw PA6 and prepared representative PA foams.

WAXD was carried out to investigate the crystalline morphologies of porous PA6 samples. The corresponding spectra of representative samples are shown in Figure 5. The main diffraction peaks are shown at 2 = 20.2° and 24.1°, attributed to the (200) and (002) crystal planes of the α crystal phase, respectively [26]. There was no crystal transformation taking place in PA foams prepared by different processing parameters. It can be concluded that there is no certain connection between the multiple melting peaks in DSC analysis and the melting of polymorphic structure of polymers.

### 3.4. Thermal Conductivity

Heat transfer within foams is composed of four distinct mechanisms [27]:

$$\lambda_{foam}=\lambda_{s}+\lambda_{g}+\lambda_{r}+\lambda_{c}$$

where *λ*_s_ and *λ*_g_ represent the thermal conductivity of the solid and the gas, respectively, *λ*_r_ is the thermal radiation term and *λ*_c_ represents the convection within the cell. *λ*_c_ can be ignored when cell size is less than 3 mm [28].

**Figure 6:**
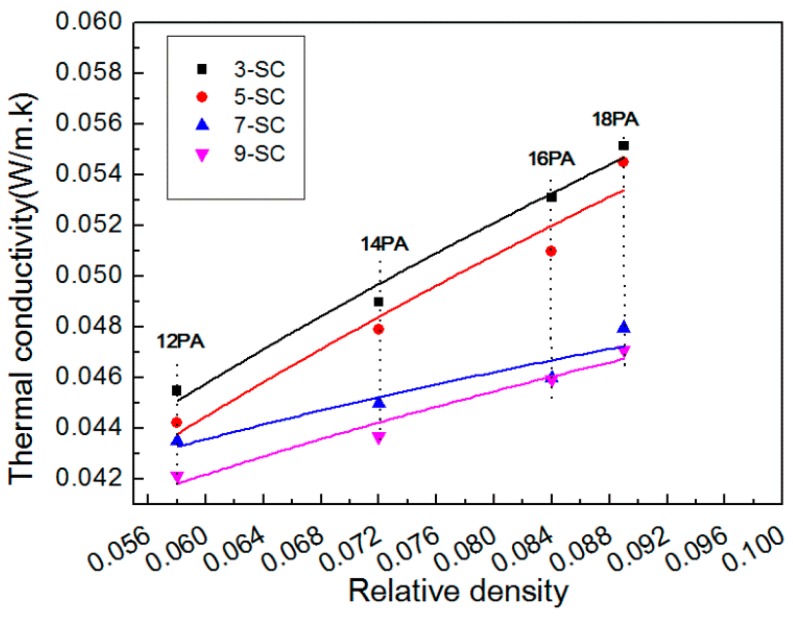
Thermal conductivity vs relative densities of PA foams.

The thermal conductivity of the porous material was very sensitive to their bulk density [29]. An increase of PA concentration increased the bulk densities of PA foams, leading to a higher contribution of *λ*_s_ and a smaller fraction of *λ*_g_ [30]. Therefore, higher thermal conductivity was obtained, as shown in Figure 6. 

As the SC concentration increased at a fixed PA content, no significant change occurred on the bulk density. However, the thermal conductivity of PA foams decreased slightly [31]. Two factors contributed to this phenomenon. First, the dimensions of pores decreased when a greater amount of SC was used, increasing the specific surface area of PA foams. As a result, the efficiency of internal gas collision and the radiation heat transfer decreased [5]. Second, higher SC concentration increased the amount of non-perfect crystals. This change of microstructure increased interfacial thermal conductivity, consuming more energy through scattering between different crystalline regions [32].

### 3.5. Mechanical Properties

**Figure 7:**
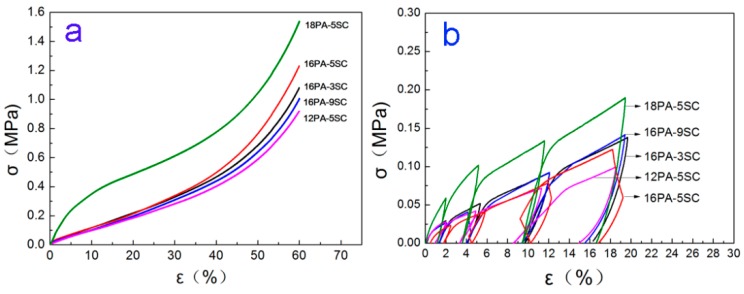
(**a**) Compressive curves of prepared PA foams and (**b**) stress–strain curves from uniaxial compression tests with intermittent unloading-reloading.

The compressive curves of prepared PA foams are shown in Figure 7a. The corresponding mechanical properties, such as compressive stress at 60% of strain (*σ*_60%_) and energy absorbed (*E*_a_), are summarized in Table 2. The energy absorbed was taken at 60% strain. Notably, the prepared foams displayed a “zero-yield-stress” phenomenon, except for 18PA-5SC, possibly due to imperfections on the end surfaces and some premature localized plastic deformation. Therefore, elastic moduli of samples were determined by a corrected method described in a previous report [33]. The corresponding stress–strain curves from uniaxial compression tests with intermittent unloading-reloading are shown in Figure 7b. The measured values of moduli (*E*) are included in Table 2.

When the PA content was 16% in solution, *E* decreased with the increase of SC content used. This can be attributed to the increased microstructural defects in pore walls caused by higher foaming power when larger quantities of SC were added. PA foams prepared from 5% SC foaming agent had the highest compressive stress (*σ*_60%_) and greatest energy absorbed (*E*_a_). This was because a more regular cellular structure was obtained for this composition. Lower or higher foaming power increased the structural defects in PA foams. When the concentration of SC increased from 3% to 5%, the cellular structures of the PA foams became more regular. More cell walls were bent when the materials were subjected to an external force. Therefore, more energy was absorbed [34]. Further increases of SC concentrations caused an excessive growth of cells, resulting in cell merger and collapse. This caused structural defects in cell walls, reducing the absorption of energy [35]. 

As PA concentration increased at 5% SC solution, PA foams with improved mechanical properties were obtained. First, bulk densities of PA foams increased as higher quantities of PA were used, resulting in thicker cell walls that could withstand higher loads. Second, the crystalline degree of materials increased due to the homogeneous nucleation effect, as mentioned in DSC analysis [36]. Moreover, the prepared PA foams displayed a hierarchal porous structure along the foaming direction as shown in Figure 3. This structure made the PA foams an excellent material for cushioning. The PA foams obtained from 18 % PA absorbed 403.7 kJ of energy at 60% of strain.

## 4. Conclusions

Porous PA materials were prepared from a PA/FA solution by a facile solution foaming strategy. The obtained PA foams showed a hierarchical porous structure along the foaming direction and the pore size ranged from 0.5 to 3 μm. By increasing the SC concentration, the foaming power and the nucleation sites increased, decreasing the pore’s dimension in foams. The size of pores also reduced as PA concentration increased because of the limited bubble expansion induced by the greater viscosity. The crystalline degree of PA foams increased with the increase of PA concentration due to the homogeneous nucleation effect. Sodium salt generated from neutralization mainly played a role of heterogeneous nucleating agent. A critical content of SC was found to produce PA foams with more regular cells and higher crystalline degree. Moreover, no crystal phase transformation occurred during the foaming process. The increase of concentration of SC solution had a minor effect on the bulk density of foams. However, it diminished the thermal conductivity of foams by increasing the interfacial thermal loss between different crystalline regions. Prepared PA foams exhibited low thermal conductivity and good mechanical properties. The novel strategy in this work could extensively produce PA foams for a range of practical applications such as thermal insulation, cushioning and adsorption, etc.

## Figures and Tables

**Table 1:** Crystalline melting temperature and crystallinity of PA foams.

Samples	*X*_1_ (%)	*X*_2_ (%)	*X* (%)	*T*_m1_ (°C)	*T*_m2_ (°C)	*T*_c1_ (°C)	*T*_c2_ (°C)
Raw PA6	25.4	/	25.4	226.3	/	165.5	/
16PA-3SC	13.6	34.7	48.3	215.9	263.9	178.2	237.9
16PA-5SC	20.9	29.8	50.7	215.5	264.3	176.1	233.4
16PA-7SC	17.1	40.2	57.3	215.8	264.0	178.5	234.3
16PA-9SC	17.3	31.6	48.9	217.8	265.3	174.0	/
12PA-5SC	24.1	29.0	53.1	216.9	265.6	176.4	227.9
14PA-5SC	9.4	45.4	54.8	216.7	264.1	179.5	233.6
18PA-5SC	26.7	35.2	61.9	216.8	264.0	177.8	240.4

**Table 2:** Compressive mechanical properties of PA foams.

Samples	*ρ*_b_ (g/cm^3^)	*E* (MPa)	*σ*_60%_ (MPa)	*E*_a_ (kJ)
16PA-3SC	0.096 ± 0.002	1.38 ± 0.15	1.08 ± 0.05	231.8 ± <0.1
16PA-5SC	0.095 ± 0.002	1.33 ± 0.10	1.23 ± 0.06	239.2 ± <0.1
16PA-7SC	0.092 ± 0.002	1.29 ± 0.07	1.12 ± 0.03	238.8 ± <0.1
16PA-9SC	0.090 ± 0.001	1.05 ± 0.06	0.96 ± 0.02	198.2 ± <0.1
12PA-5SC	0.066 ±< 0.001	1.02 ± 0.07	0.92 ± 0.02	199.5 ± <0.1
14PA-5SC	0.082 ± 0.001	1.14 ± 0.04	1.21 ± 0.04	237.1 ± <0.1
18PA-5SC	0.101 ±<0.001	4.74 ± 0.08	1.53 ± 0.05	403.7 ± <0.1

## References

[1] Wypych G., Wypych G. (2012). Polyamide 6. Handbook of Polymers.

[2] Schüler F., Schamel D., Salonen A., Drenckhan W., Gilchrist M.D., Stubenrauch C. (2012). Synthesis of macroporous polystyrene by the polymerization of foamed emulsions. Angew. Chem. Int. Ed..

[3] Mills N.J. (2007). Polymer Foams Handbook.

[4] Colton J.S., Suh N.P. (2010). Nucleation of microcellular foam: Theory and practice. Polym. Eng. Sci..

[5] Liu S., Duvigneau J., Vancso G.J. (2015). Nanocellular polymer foams as promising high performance thermal insulation materials. Eur. Polym. J..

[6] Moglia R., Whitely M., Dhavalikar P., Robinson J., Pearce H., Brooks M., Stuebben M., Cordner N., Cosgriffhernandez E. (2014). Injectable polymerized high internal phase emulsions with rapid in situ curing. Biomacromolecules.

[7] Lumelsky Y., Zoldan J., Levenberg S., Silverstein M.S. (2008). Porous polycaprolactone-polystyrene semi-interpenetrating polymer networks synthesized within high internal phase emulsions. Macromolecules.

[8] Tai H., Sergienko A., Silverstein M.S. (2001). Organic-inorganic networks in foams from high internal phase emulsion polymerizations. Polymer.

[9] Rahman M.A., Renna L.A., Venkataraman D., Desbois P., Lesser A.J. (2018). High crystalline, porous polyamide 6 by anionic polymerization. Polymer.

[10] Wang L., Schiraldi D.A., Sanchez-Soto M. (2014). Foam-like xanthan gum/clay aerogel composites and tailoring properties by blending with agar. Ind. Eng. Chem. Res..

[11] Wang L., Sánchez-Soto M., Maspoch M.L. (2013). Polymer/clay aerogel composites with flame retardant agents: Mechanical, thermal and fire behavior. Mater. Des..

[12] Rudaz C., Courson R., Bonnet L., Calasetienne S., Sallée H., Budtova T. (2014). Aeropectin: Fully biomass-based mechanically strong and thermal superinsulating aerogel. Biomacromolecules.

[13] Grishechko L.I., Amaral-Labat G., Szczurek A., Fierro V., Kuznetsov B.N., Pizzi A., Celzard A. (2013). New tannin-lignin aerogels. Ind. Crop. Prod..

[14] Kwon J., Kim J., Park D., Han H. (2015). A novel synthesis method for an open-cell microsponge polyimide for heat insulation. Polymer.

[15] Verdolotti L., Lavorgna M., Lamanna R., Di Maio E., Iannace S. (2015). Polyurethane-silica hybrid foam by sol-gel approach: Chemical and functional properties. Polymer.

[16] Rafique F.Z., Vasanthan N. (2014). Crystallization, crystal structure, and isothermal melt crystallization kinetics of novel polyamide 6/SiO_2_ nanocomposites prepared using the sol-gel technique. J. Phys. Chem. B.

[17] Seo W.J., Park J.H., Sung Y.T., Hwang D.H., Kim W.N., Lee H.S. (2010). Properties of water-blown rigid polyurethane foams with reactivity of raw materials. J. Appl. Polym. Sci..

[18] Realinho V., Haurie L., Antunes M., Velasco J.I. (2014). Thermal stability and fire behaviour of flame retardant high density rigid foams based on hydromagnesite-filled polypropylene composites. Compos. Part B.

[19] Zhang D., Burkes W.L., Schoener C.A., Grunlan M.A. (2012). Porous inorganic-organic shape memory polymers. Polymer.

[20] Yu E., Ikeya M., Okamoto M. (2006). Foam processing and cellular structure of polylactide-based nanocomposites. Polymer.

[21] Bian J., Ye S.R., Feng L.X. (2003). Heterogeneous nucleation on the crystallization poly(ethylene terephthalate). J. Polym. Sci. Part B Polym. Phys..

[22] Abbasi H., Antunes M., Velasco J.I. (2018). Effects of carbon nanotubes/graphene nanoplatelets hybrid systems on the structure and properties of polyetherimide-based foams. Polymers.

[23] Huang Z., Yin Q., Wang Q., Wang P., Liu T., Qian L. (2017). Mechanical properties and crystallization behavior of three kinds of straws/nylon 6 composites. Int. J. Boil. Macromol..

[24] Delkash M., Naderi G., Sahraieyan R., Esmizadeh E. (2017). Crystallization, structural and mechanical properties of PA6/PC/NBR ternary blends: Effect of NBR-g-GMA compatibilizer and organoclay. Sci. Eng. Compos. Mater..

[25] Diao J., Salazar R., Kelton K.F., Gelb L.D. (2008). Impact of diffusion on concentration profiles around near-critical nuclei and implications for theories of nucleation and growth. Acta Mater..

[26] Shan G.F., Yang W., Tang X.G., Yang M.B., Xie B.H., Fu Q., Mai Y.W. (2010). Multiple melting behaviour of annealed crystalline polymers. Polym. Test..

[27] Antonio R.R.J., Cristina S.A., Michel D., Rodríguez-Perez M.A., Leo G. (2011). Production, cellular structure and thermal conductivity of microcellular (methyl methacrylate)-(butyl acrylate)-(methyl methacrylate) triblock copolymers. Polym. Int..

[28] Notario B., Pinto J., Solorzano E., Saja J.A.D., Dumon M., Rodríguez-Pérez M.A. (2015). Experimental validation of the knudsen effect in nanocellular polymeric foams. Polymer.

[29] Solórzano E., Reglero J.A., Rodríguez-Pérez M.A., Lehmhus D., Wichmann M., Saja J.A.D. (2008). An experimental study on the thermal conductivity of aluminium foams by using the transient plane source method. Int. J. Heat Mass Transf..

[30] Luo Y., Ye C. (2012). Using nanocapsules as building blocks to fabricate organic polymer nanofoam with ultra low thermal conductivity and high mechanical strength. Polymer.

[31] Qiu L., Zheng X.H., Zhu J., Tang D.W., Yang S.Y., Hu A.J., Wang L.L., Li S.S. (2015). Thermal transport in high-strength polymethacrylimide (pmi) foam insulations. Int. J. Thermophys..

[32] Saruhan B., Ryukhtin V., Kelm K. (2011). Correlation of thermal conductivity changes with anisotropic nano-pores of eb-pvd deposited fysz-coatings. Surf. Coat. Technol..

[33] Sun Y., Amirrasouli B., Razavi S.B., Li Q.M., Lowe T., Withers P.J. (2016). The variation in elastic modulus throughout the compression of foam materials. Acta Mater..

[34] Sotomayor O.E., Tippur H.V. (2014). Role of cell regularity and relative density on elasto-plastic compression response of random honeycombs generated using voronoi diagrams. Int. J. Solids Struct..

[35] Root S., Haill T.A., Lane J.M.D., Thompson A.P., Grest G.S., Schroen D.G., Mattsson T.R. (2013). Shock compression of hydrocarbon foam to 200 gpa: Experiments, atomistic simulations, and mesoscale hydrodynamic modeling. J. Appl. Phys..

[36] Jaeger P.D., Huisseune H., Ameel B., Paepe M.D. (2011). An experimentally validated and parameterized periodic unit-cell reconstruction of open-cell foams. J. Appl. Phys..

